# Correlation analysis between body composition, serological indices and the risk of falls, and the receiver operating characteristic curve of different indexes for the risk of falls in older individuals

**DOI:** 10.3389/fmed.2023.1228821

**Published:** 2023-07-25

**Authors:** Kexin Zhang, Yanmin Ju, Di Yang, Mengyu Cao, Hong Liang, Jiyan Leng

**Affiliations:** Department of Cadre Ward, The First Hospital of Jilin University, Changchun, China

**Keywords:** older adults, comprehensive geriatric assessment, body composition, serological indices, fall risk

## Abstract

**Objective:**

This study assessed the risk factors for falls and evaluated the correlation between body composition, serological indices, and the risk of falls in older individuals.

**Method:**

This cross-sectional study included 387 individuals ≥60 years of age in the cadre ward of the First Hospital of Jilin University. The information used in this study was obtained from the comprehensive geriatric assessment database of the cadre ward. The body composition of the individuals was measured by bioelectrical impedance analysis using an InBody S10 device. We assessed fall risk using the fall risk assessment tool. Individuals with ≤2 points were placed in the low-risk group, those with 3–9 points were placed in the medium-risk group, and those with ≥10 points were placed in the high-risk group.

**Results:**

Differences in age, educational background, height, cognitive impairment, malnutrition, ability of daily living, depression, diastolic blood pressure, heart rate, intracellular water, total body moisture, water ratio, limb moisture (right and left, upper and lower), trunk moisture, fat-free weight, arm girth, body cell mass, skeletal muscle mass, limb muscle (right and left, upper and lower), appendicular skeletal muscle mass index (ASMI), sarcopenia, hemoglobin level, hematocrit level, aspartate aminotransferase level, albumin level, anemia, and hypoproteinemia were observed among the three groups (*p* < 0.001, *p* = 0.002, *p* = 0.006, *p* < 0.001, *p* < 0.001, *p* < 0.001, *p* < 0.001, *p* < 0.001, *p* = 0.008, *p* = 0.010). Ordinal logistic regression analysis showed that the probability of the fall risk increasing by one level was 1.902 times higher for each unit of decrease in educational background, respectively. In addition, the probability of the fall risk increasing by one level was 2.971, 3.732, 3.804, 1.690 and 2.155 times higher for each additional unit of age, cognitive impairment, lower limb edema, decreased skeletal muscle mass, and sarcopenia, respectively.

**Conclusion:**

Our findings suggest that educational background, age, cognitive impairment, lower limb edema, decreased skeletal muscle mass, and sarcopenia were associated with falls in older individuals. Body composition and serological indices can assist in the early identification of falls in the older people.

## 1. Introduction

At present, China has entered a society with an aging population. Research shows that, by the end of 2018, China’s older population aged 60 and over had reached 249 million, accounting for 17.9% of the total population in the country (1). Research has shown that approximately 30% of people >65 years-of-age falls every year. The injury caused by falls is much more severe than that caused by other factors (2, 3).

Falls are an important cause of disability and death in the older population and are defined as sudden, involuntary, and unintentional body position changes in individuals, resulting in body parts other than feet making contact with the ground or surfaces lower than the ground (4). Falls are the most common health problem among older adults. Falls can lead to soft tissue injury and fracture in older individuals and even death in severe cases (5). The incidence of falls in older adults is high. According to epidemiological data, the incidence of falls in Chinese residents aged >60 years is high and the incidence of falls increases with age (6). In the case of fractures and soft tissue contusions after falling, individuals require bed rest for long periods of time, which increases the risk of bedsores, deep vein thrombosis, and accumulated pneumonia, further affecting their quality of life.

Therefore, it is necessary to identify potential risk factors for falls in older people and formulate targeted fall prevention programs. On this basis, our research team carried out a comprehensive assessment of older people in the cadre ward of the First Hospital of Jilin University, taking the older population as the research object, in order to further explore the potential correlation between older people’s body composition, serological indicators and falls, and to provide relevant schemes for the elderly to prevent falls.

## 2. Materials and methods

### 2.1. Participants and eligibility criteria

The participants were individuals ≥60 years of age in the cadre ward of the First Hospital of Jilin University from May 1, 2019, to December 31, 2022. The inclusion criteria were as follows: (1) age ≥ 60 years and (2) completion of the comprehensive assessment questionnaire and test for older adults. The exclusion criteria were as follows: (1) lack of basic data and indicators of the comprehensive assessment questionnaire for older adults and (2) suffering from malignant tumors, immune system diseases, serious liver and kidney dysfunction, and other serious diseases.

### 2.2. Measurement

The medical records of all individuals who met the eligibility criteria at the First Hospital of Jilin University from May 1, 2019, to December 31, 2022, were used in this study. The medical information used included height, weight, sex, age, education, smoking history, drinking history, and serological indices, such as percentage of neutrophils, absolute neutrophil count, hemoglobin, hematocrit, aspartate aminotransferase, alanine aminotransferase, alkaline phosphatase, cholinesterase, albumin, prealbumin, cholesterol, triglyceride, high-density lipoprotein cholesterol, low-density lipoprotein cholesterol, and fasting blood glucose levels. A Jamar handgrip dynamometer was used to measure grip strength. The 4-m pace of individuals, measured using a stopwatch, was obtained from the past comprehensive geriatric assessment database of the cadre ward. The body composition of the individuals was measured by bioelectrical impedance analysis (BIA) using an Inbody S10 device (Biospace, Seoul, Republic of Korea). In addition, the individuals were assessed using the Activities of Daily Living Scale (ADL and IADL), Mini–Mental State Examination (MMSE), Geriatric Depression Scale (GDS), Falls Risk Assessment (FRA), and Mini Nutrition Assessment Scale (MNA) (Part I, Part I + Part II).

### 2.3. Body composition analysis

The body composition of the individuals was measured by BIA using an InBody S10 device (Biospace). All individuals were placed in the supine position for 10 min before analysis. During the analysis, the individuals were placed in the supine position with arms abducted at 15°, starting from the torso and legs, and separated by shoulder width. Eight electrodes were placed on the hands (thumb and middle fingers), ankles, and heels of the individuals. Alcohol was used to clean the skin before placing the electrodes to maximize skin contact. Patient age, sex, height, and weight were used as inputs to measure the body composition. All measurements in this study were performed by the same physician.

### 2.4. Fall risk assessment

The fall risk assessment tool (FRA) was used in this study. This scale was released in 2011 by the Ministry of Health of China (7), including 8 dimensions of exercise, history of falls, unstable mental state, self-control ability, sensory disorders, sleep status, medication history, and related medical history. A total of 35 items were included in the assessment; each item had 1–3 points, and the total score of the scale was 53 points. Participants with ≤2 points were placed in the low-risk group, those with 3–9 points in the medium-risk group, and those with ≥10 points in the high-risk group. It takes 5–10 min to complete the entire scale. In 2014, Zhuge Yi et al. (8) used the revised fall efficacy scale from abroad as a reference (9) and applied the guideline scale to assess the risk of falls in the elderly. This scale has good reliability and validity and can be used to screen for the risk of falls in older Chinese adults.

### 2.5. Other assessments

#### 2.5.1. Activities of Daily Living Scale

ADL and IADL scale includes the most basic aspects of daily life (10), such as clothing, food, housing, transportation, self-care, and social behavior. There are 14 items in total, divided into 4 levels: 1. You can do it yourself; 2- Some difficulties; 3- Need help; 4- There’s no way to do it. The total score is 56 points, with a minimum score of 14 points (11). Divided into 2 subscales, namely Physical Activity of Daily Living (PADL) and Instrumental Activity of Daily Living (IADL), with 7 items each. The scale operation is relatively simple and the completion time is short. The patient and their family members can objectively and smoothly cooperate with the staff to complete the assessment.

#### 2.5.2. Nutritional status

The MNA (12) has been used in previous studies to assess the nutritional status of older adults and as an evidence-based screening tool of older adults. MNA scores <17 indicated malnutrition in the participants.

#### 2.5.3. Depression

The GDS screening questionnaire (13) was specifically designed for older adults. The GDS has a total of 30 items, each of which has one point, and the total score of the scale is 30 points. Participants with ≤10 points were considered normal, while participants with 11–20 points were considered to have mild depression, and those with 21–30 points were considered to have moderate-to-severe depression (14). In this study, scores of >10 points were defined as abnormal.

#### 2.5.4. Cognitive function

MMSE is a psychometric screening tool used to assess cognitive function. The boundary of cognitive normality was defined according to the level of education of individuals, with primary school education being >20 points, junior high school or above being >24 points, and below these levels of education defined as cognitive dysfunction. Gao et al. (15) evaluated the influencing factors of MMSE scores and the screening validity of normal values. The MMSE test results of 19,117 normal elderly people and 137 dementia patients were compared with the screening validity of various critical values of MMSE using the area and validity indicators under the working characteristic curve. Multiple linear regression was used to compare the differences in influencing factors of MMSE scores between normal and dementia populations. The research showed that MMSE has good reliability and validity and can be used to screen for depression in older Chinese adults.

#### 2.5.5. Assessment of sarcopenia

Sarcopenia was diagnosed according to consensus of the Asian Working Group for Sarcopenia in 2019 (16): (1) the walking speed cut point was a walking speed ≤0.8 m/s; and (2) the grip strength cut point was <26 kg for men and < 18 kg for women. When (1) and/or (2) was found, muscle mass, including skeletal muscle mass of the limbs and the whole body, of the patient was further measured by bioelectrical impedance analysis, and the appendicular skeletal muscle mass index (ASMI) was calculated. Patients with an ASMI <7.0 kg/m^2^ (men) or < 5.7 kg/m^2^ (women) were diagnosed with sarcopenia.

### 2.6. Statistical methods

Statistical analyses were performed using the SPSS statistical software (version 26.0; IBM Corp., Armonk, NY, USA). Continuous variables are presented as mean ± standard deviation (SD) or median (interquartile range) based on the distribution characteristics of the data. The classified variables were analyzed using percentages. The quantitative data of the two groups were compared using *t-*tests, and multiple groups were compared using one-way ANOVA. Indicators such as sex were compared using chi-square (χ^2^) tests; if the data did not conform to a normal distribution, rank sum tests were used. Ordinal logistic regression analysis was used to determine the correlations between body composition, serological indices, and fall risk. Te AUC value was calculated by drawing ROC curve to identify the association between different indexes and the risk of falls. The Youden index was used to determine the optimal cutoff point. All statistical tests were conducted using a two-sided, and differences were considered statistically significant at *p* < 0.05.

## 3. Results

### 3.1. Baseline characteristics

As shown in Supplementary Table S1, a total of 387 individuals in the study. Differences in age, educational background, height, cognitive impairment, malnutrition, ability to perform daily living, depression, MMSE scores, heart rate and diastolic blood pressure between the three groups were statistically significant (*p* < 0.001, *p* = 0.002, *p* = 0.006, *p* < 0.001, *p* < 0.001, *p* < 0.001, *p* < 0.001, *p* < 0.001, *p* = 0.008, *p* = 0.010).

### 3.2. Comparison of body composition among low-, medium-, and high-risk groups

As shown in Supplementary Table S2, differences were observed in intracellular water, total body moisture, water ratio, right and left upper limb moisture, trunk moisture, right and left lower limb moisture, lower limb edema, fat-free weight, arm girth, body cell mass, skeletal muscle mass, right and left upper limb muscle, right and left lower limb muscle, appendicular skeletal muscle mass index (ASMI) and sarcopenia among the three groups (*p* < 0.05). The high-risk group had higher water ratio, trunk moisture, limb moisture, lower limb edema, sarcopenia, lower intracellular water, total body moisture, fat-free weight, arm girth, body cell mass, skeletal muscle mass, limb muscle, ASMI than the other two groups. The differences listed above were statistically significant (*p* < 0.05).

### 3.3. Comparison of serological indices among low-, medium-, and high-risk groups

There were differences between the groups in hemoglobin (HGB), hematocrit (HCT), aspartate aminotransferase (AST), albumin (ALB), and the presence of anemia and hypoproteinemia in Supplementary Table S3 (*p* < 0.05). The high-risk group had lower HGB, HCT, ALT, ALB levels, and anemia and hypoproteinemia were more severe in this group than in the other two groups. The differences listed above were statistically significant (*p* < 0.05).

### 3.4. Ordinal logistic regression analysis of body composition and serological indices of the low-, medium-, and high-risk groups to predict the risk of falls

Supplementary Table S4 showed that the probability of the fall risk increasing by one level was 1.902 times higher for each unit of decrease in educational background, respectively (OR = 0.526, 95% CI: 0.283–0.975, *p* = 0.042). In addition, the probability of the fall risk increasing by one level was 2.971, 3.732, 3.804, 1.690, and 2.155 times higher for each additional unit of age, cognitive impairment, lower limb edema, decreased skeletal muscle mass, and sarcopenia, respectively (OR = 2.971, 95% CI: 1.667–5.296, *p* < 0.001; OR = 3.732, 95% CI: 1.438–9.689, *p* = 0.007; OR = 3.804, 95% CI: 1.655–8.741, *p* = 0.002; OR = 1.690, 95% CI: 1.015–2.818, *p* = 0.044; OR = 2.155, 95% CI: 1.186–3.916, *p* = 0.012).

### 3.5. The association of different indexes with the occurrence of falls

**Figure 1:**
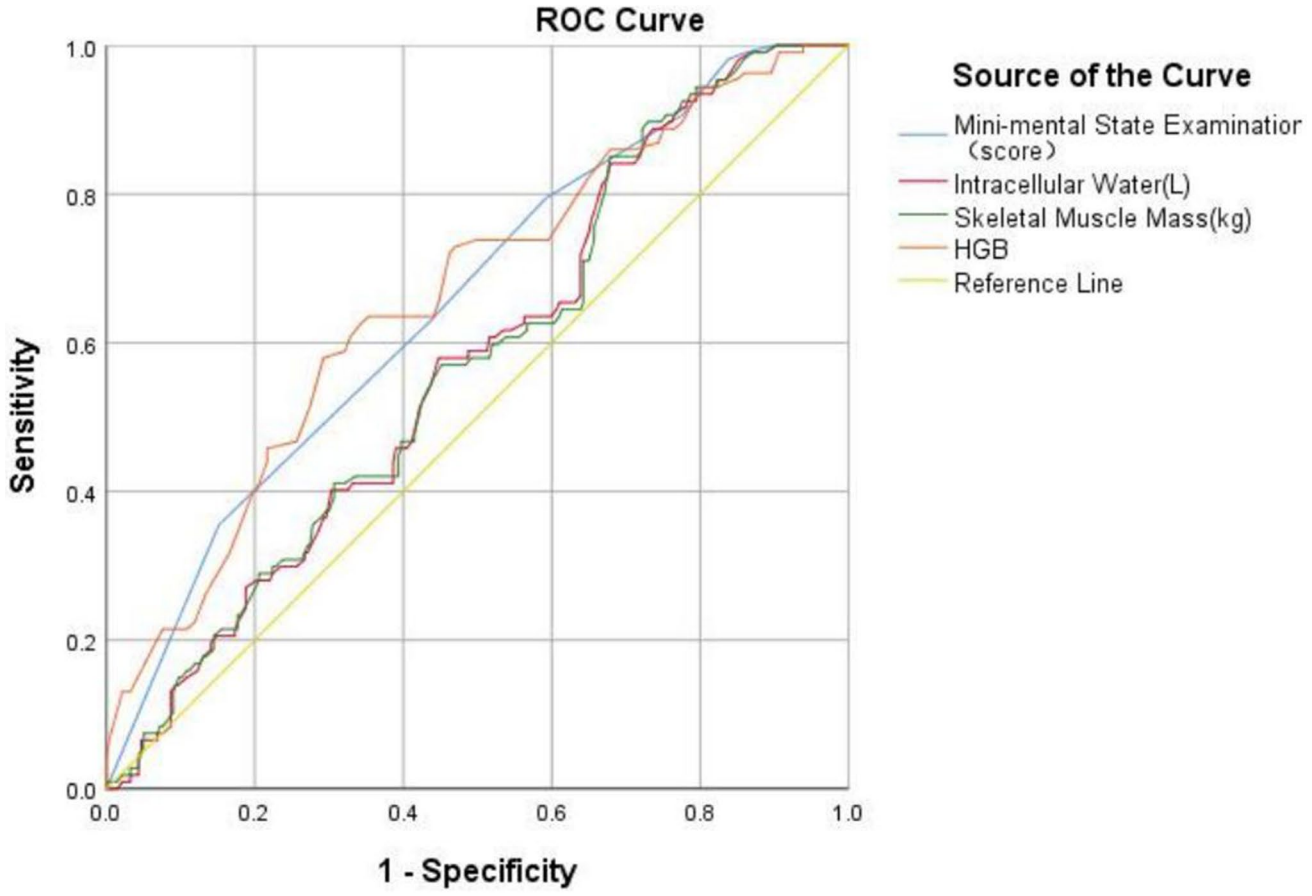
The receiver operating characteristics curve of different indexes for the occurrence of falls.

The correlation between different indexes on the occurrence of falls in older people was analyzed and compared. Results are shown in Supplementary Table S5, the area under the curve of MMSE score, intracellular water, skeletal muscle mass and hemoglobin was larger (AUC > 0.5), AUC was 0.654, 0.574, 0.574, and 0.663, respectively. However, through the comparison, it was found that hemoglobin exhibited weakly superiority for the occurrence of falls than MMSE score, intracellular water, skeletal muscle mass. The relevant ROC curve is shown in Figure 1.

## 4. Discussion

Falls in older individuals are not only a serious social problem, but also an urgent medical problem. In our study, we found that limb moisture, fat-free weight, arm girth, skeletal muscle mass, limb muscle mass, HGB, and hypoproteinemia are closely related to the risk of falls in the older adult population. Additionally, educational background and hemoglobin level were significant protective factors, while age, cognitive impairment, malnutrition, and limb moisture were significant risk factors for falls. According to the results obtained in our study, such as body composition and serological indices, appropriate fall prevention programs can be developed, and the safety and property of older individuals can be preserved.

This study found that the fall risk gradually increased with age, which is consistent with the findings of Timsina et al. (17). Although falls can occur in any age group, the fall risk in older individuals is higher than that in younger individuals (18).

In our research, the incidence of falls was higher in older individuals with low educational levels, poor ability in daily living and depressive states. This result suggests that the occurrence of falls is related to education level, which may be due to the strong health awareness of individuals with high educational levels. Many studies have found that older individuals with cognitive impairment are more likely to fall (19, 20), possibly because when cognitive ability decreases, the speed and executive ability to obtain and process external information is reduced to varying degrees, which increases the possibility of falls. Simultaneously, poor ability in daily living leads to a decline in adaptability to the environment, further increasing the possibility of falls. The results of this study is consistent with the above view. Depressive states not only lead to loneliness and depression but also reduce attention to the external environment. This decline in executive ability affects their ability to complete dual tasks simultaneously, such as walking and speaking, and to fully process information, which further causes gait and balance problems, leading to falls or affecting recovery after falls (21).

This study analyzed the correlations among serum indicators, nutritional status, and falls. The results showed that hemoglobin and albumin levels, malnutrition, and fall risk were closely related. Decreased hemoglobin levels increase the risk of anemia, fatigue, and falls. Hemoglobin carries oxygen; therefore, decreases in oxygenated hemoglobin levels result in an insufficient oxygen supply to muscle tissues and the brain. Changing body position increases the risk of falls (22). In a previous multicenter cohort study of older adult males in the United States, a decrease in albumin levels indicated a reduced possibility of improving asthenia. Albumin level is an important factor affecting the prognosis of older adults (23). Albumin levels reflect nutritional status to some extent (24). Many studies have shown that frailty is related to falls, disability, and increased risk of death (25–27).

In this study, we analyzed the comparison of body composition among the three risk groups, we found that limb moisture, fat-free weight, arm girth, skeletal muscle mass, limb muscle mass, are closely related to the risk of falls in the older adult population. Additionally, limb moisture were significant risk factors for falls. The reduction in muscle mass reduces the types of motor neurons present, which will have an impact on muscle performance, muscle quality, and strength, affecting the walking speed, walking morphology, and balance ability of older individuals (28, 29) and increasing the risk of falls (30, 31). Therefore, a decrease in muscle mass, especially skeletal muscle mass of the limbs, is a risk factor for falls. Many studies (32, 33) have shown that individuals with heart failure are often accompanied by frailty. To understand the occurrence of disability in individuals with cardiovascular disease. Afilolo et al. (34) summarized and analyzed the data of nine studies which included 54,250 community residents and concluded that the incidence of disability in older adults individuals with cardiovascular disease was 25–50%. Nadruz et al. (35) showed that older individuals are generally prone to frailty after suffering from heart failure. In addition, lower limb moisture often indicates the occurrence and severity of heart failure, and there is a close relationship between frailty and fall risk.

The results of this study highlight the need to actively publicize the importance of preventing falls in the older adult population, conducting regular examinations, planning meals, and increasing protein intake, which are conducive to strengthening muscle mass, protecting body balance, and reducing the risk of falls.

This study had some limitations. First, falls are complex outcomes caused by several factors, and there are many factors related to falls, such as hearing, vision, gait, and lower limb muscle strength, which were not included in this study. Therefore, the status of the participants could not be comprehensively evaluated. In conclusion, more prospective cohort studies are needed to further understand the potential role of body composition and serological indices in preventing falling of older people.

## 5. Conclusion

Our findings suggest that educational background, age, cognitive impairment, lower limb edema, decreased skeletal muscle mass, and sarcopenia were associated with falls in older individuals. Body composition and serological indices can assist in the early identification of falls in the older people.

## Data availability statement

The original contributions presented in the study are included in the article/Supplementary material, further inquiries can be directed to the corresponding author.

## Ethics statement

The studies involving human participants were reviewed and approved by the First Hospital of Jilin University. The patients/participants provided their written informed consent to participate in this study.

## Author contributions

KZ, YJ, DY, MC, HL, and JL were involved in the conceptualization and design of the study, data analysis and interpretation, and manuscript preparation. KZ, YJ, DY, MC, and HL acquired participants and data. All authors contributed to the article and approved the submitted version.

## Funding

Funding for this study was received through the science and technology development plan project “Intelligent evaluation and management platform for older adults health status” (Project No.: 20200404207yy) of the Department of science and technology of Jilin Province; “Epidemiological investigation of senile syndrome and predictive value of machine learning” (Project No.: 2022C043-7) of the Jilin Provincial Development and Reform Commission; and “Study on related factors of sarcopenia in older adults individuals with heart failure” (Project No.: JLSWSRCZX2021-046) of the Jilin Provincial Department of Finance.

## Conflict of interest

The authors declare that the research was conducted in the absence of any commercial or financial relationships that could be construed as a potential conflict of interest.

## Publisher’s note

All claims expressed in this article are solely those of the authors and do not necessarily represent those of their affiliated organizations, or those of the publisher, the editors and the reviewers. Any product that may be evaluated in this article, or claim that may be made by its manufacturer, is not guaranteed or endorsed by the publisher.
