# Supplementary material for: Correlation analysis between body composition, serological indices and the risk of falls, and the receiver operating characteristic curve of different indexes for the risk of falls in older individuals
Source: Front Med (Lausanne). 2023 Jul 25;10:1228821. doi: 10.3389/fmed.2023.1228821 (PMC10409486; doi:10.3389/fmed.2023.1228821)
Supplement: Supplementary file 1 [file Table_1.DOCX]

Supplementary Material

Correlation Analysis Between Body Composition, Serological Indices and the Risk of Falls and **the receiver operating characteristic curve of different indexes for** the Risk of Falls in Older Individuals.

Kexin Zhang^1^, Yanmin Ju^1^, Di Yang^1^, Mengyu Cao^1^, Hong Liang^1^, Jiyan Leng^1*^

^1^Department of Cadre ward, The First Hospital of Jilin University, Changchun 130021, China

*** Correspondence:**Jiyan Leng
lengjy@jlu.edu.cn

# Supplementary Tables

**Table1** Comparison of demographic information of the individuals

|  | **Low Risk Group（N=107）** | **Medium Risk Group（N=218）** | **High risk Risk Group（N=62）** | **P** |
| --- | --- | --- | --- | --- |
| **Age（years old）** |  |  |  | <0.001^*^ |
| **60-69** | 56（52.3%） | 80（36.7%） | 4（6.5%） |  |
| **70-79** | 30（28.0%） | 50（22.9%） | 11（17.7%） |  |
| **≥80** | 21（19.6%） | 88（40.4%） | 47（75.8%） |  |
| **Gender (Male)** | 76（71.0%） | 152（69.7%） | 38（61.3%） | 0.375 |
| **Education** |  |  |  | 0.002^*^ |
| **Illiteracy** | 23 (21.5%) | 52 (23.9%) | 8 (12.9%) |  |
| **Primary School** | 4 (3.7%) | 21 (9.6%) | 8 (12.9%) |  |
| **Middle School** | 11 (10.3%) | 35 (16.1%) | 19 (30.6%) |  |
| **College degree or above** | 69 (64.5%) | 110 (50.5%) | 27 (43.5%) |  |
| **Height（cm）** | 167.9±7.2 | 157.9±40.1^a^ | 149.9±49.6^a^ | 0.006^*^ |
| **Weight（Kg）** | 70.7±11.3 | 69.3±14.0 | 66.4±10.7^a^ | 0.104 |
| **Smoking (Yes)** | 68 (63.6%) | 142 (65.4%) | 46 (74.2%) | 0.086 |
| **Alcohol (Yes)** | 63 (58.9%) | 134 (61.5%) | 43 (69.3%) | 0.115 |
| **Cognitive Impairment (Yes)** | 0（0.0%） | 14（6.4%） | 14（22.6%） | <0.001^*^ |
| **Malnutrition (Yes)** | 4（3.7%） | 30（13.8%） | 18（29.0%） | <0.001^*^ |
| **Ability of Daily Living（score）** | 14.7±1.3 | 15.7±3.2 | 22.1±8.8^ab^ | <0.001^*^ |
| **Depressive State（score）** | 18.8±11.3 | 19.6±12.9 | 26.3±13.0^ab^ | <0.001^*^ |
| **Mini-mental State Examination（score）** | 28.7±1.4 | 27.7±2.5^a^ | 25.6±5.0^ab^ | <0.001^*^ |
| **Systolic Pressure（mmHg）** | 133.4±16.8 | 135.9±23.6 | 139.6±17.7 | 0.182 |
| **Diastolic Pressure（mmHg）** | 77.8±10.4 | 76.5±12.0 | 72.4±10.2^ab^ | 0.010^*^ |
| **Heart Rate（cpm）** | 73.2±9.9 | 68.9±12.9^a^ | 70.2±10.3 | 0.008^*^ |

^a^ indicates that the difference was statistically significant compared to the low-risk group.

^b^ indicates that the difference is statistically significant compared to the medium-risk group.

^*^ P< 0.05.
