# Supplementary material for: Correlation analysis between body composition, serological indices and the risk of falls, and the receiver operating characteristic curve of different indexes for the risk of falls in older individuals
Source: Front Med (Lausanne). 2023 Jul 25;10:1228821. doi: 10.3389/fmed.2023.1228821 (PMC10409486; doi:10.3389/fmed.2023.1228821)
Supplement: Supplementary file 2 [file Table_2.DOCX]

Supplementary Material

Correlation Analysis Between Body Composition, Serological Indices and the Risk of Falls and **the receiver operating characteristic curve of different indexes for** the Risk of Falls in Older Individuals.

Kexin Zhang^1^, Yanmin Ju^1^, Di Yang^1^, Mengyu Cao^1^, Hong Liang^1^, Jiyan Leng^1*^

^1^Department of Cadre ward, The First Hospital of Jilin University, Changchun 130021, China

*** Correspondence:**Jiyan Leng
lengjy@jlu.edu.cn

# Supplementary Tables

**Table2** Comparison of body composition among the low-, medium-, and high-risk groups

|  | **All** | **Low Risk Group** | **Medium Risk Group** | **High risk Risk Group** | **P** |
| --- | --- | --- | --- | --- | --- |
| **Intracellular Water(L)** | 21.2±4.0 | 21.9±3.5 | 21.3±4.2 | 19.5±3.7^ab^ | 0.001^*^ |
| **Extracellular Water(L)** | 13.5±2.4 | 13.7±2.2 | 13.6±2.6 | 13.1±2.5 | 0.215 |
| **Total Body Moisture(L)** | 34.7±6.4 | 35.6±5.7 | 34.9±6.7 | 32.6±6.1^ab^ | 0.009^*^ |
| **Water Ratio** | 0.390±0.011 | 0.385±0.008 | 0.390±0.011^a^ | 0.400±0.010^ab^ | <0.001^*^ |
| **Right Upper Limb Moisture(L)** | 0.378±0.012 | 0.376±0.011 | 0.378±0.014 | 0.382±0.006^ab^ | 0.025^*^ |
| **Left Upper Limb Moisture(L)** | 0.384±0.022 | 0.378±0.012 | 0.379±0.010 | 0.405±0.039^ab^ | 0.005^*^ |
| **Trunk Moisture(L)** | 0.391±0.013 | 0.386±0.009 | 0.391±0.012^a^ | 0.401±0.012^ab^ | <0.001^*^ |
| **Right Lower Limb Moisture(L)** | 0.390±0.013 | 0.385±0.010 | 0.389±0.013^a^ | 0.402±0.011^ab^ | <0.001^*^ |
| **Left Lower Limb Moisture(L)** | 0.394±0.013 | 0.388±0.010 | 0.393±0.012^a^ | 0.406±0.011^ab^ | <0.001^*^ |
| **Lower limb Edema (Yes)** | 244 (63.0%) | 43 (40.2%) | 143 (65.6%) | 58 (93.5%) | <0.001^*^ |
| **Fat Free Weight(kg)** | 47.2±8.7 | 48.4±7.8 | 47.5±9.1 | 44.1±8.3^ab^ | 0.006^*^ |
| **Body Fat(kg)** | 21.7±7.9 | 22.2±7.6 | 21.3±8.1 | 22.2±7.7 | 0.532 |
| **Waistline(cm)** | 80.3±12.4 | 80.1±13.2 | 80.8±12.3 | 79.0±11.3 | 0.616 |
| **Arm Girth(cm)** | 28.7±4.9 | 29.8±7.4 | 28.5±3.6^a^ | 27.9±2.6^a^ | 0.016^*^ |
| **Body Cell Mass(kg)** | 30.4±5.8 | 31.3±5.1 | 30.6±6.0 | 28.0±5.3^ab^ | 0.001^*^ |
| **BMI(kg/m^2^)** | 24.7±3.6 | 25.0±3.0 | 24.7±3.9 | 24.5±3.7 | 0.634 |
| **Skeletal Muscle Mass(kg)** | 25.9±6.5 | 27.2±8.4 | 25.9±5.5 | 23.5±4.9^ab^ | 0.001^*^ |
| **Body Fat Percentage(%)** | 31.2±8.8 | 31.0±7.7 | 30.8±9.3 | 33.2±8.5 | 0.158 |
| **Visceral Fat Area(cm^2^)** | 89.0±40.9 | 85.8±38.0 | 88.4±43.6 | 96.6±35.3 | 0.241 |
| **Arm Muscle Dimension(cm)** | 23.8±5.8 | 23.5±3.9 | 24.1±5.5 | 23.3±8.7 | 0.519 |
| **Bone Mineral Content(kg)** | 2.8±1.6 | 2.8±0.5 | 2.9±2.0 | 2.7±1.0 | 0.692 |
| **Right Upper Limb Muscle(kg)** | 2.0±0.7 | 2.2±0.7 | 2.2±0.7 | 1.9±0.6^ab^ | 0.024^*^ |
| **Left Upper Limb Muscle(kg)** | 2.1±0.7 | 2.2±0.6 | 2.1±0.7 | 1.8±0.5^ab^ | 0.003^*^ |
| **Trunk Muscle(kg)** | 19.2±5.2 | 19.4±4.3 | 19.3±4.4 | 18.4±8.4 | 0.429 |
| **Right Lower Limb Muscle(kg)** | 8.7±2.1 | 9.0±1.9 | 8.7±2.0 | 8.1±1.9^ab^ | 0.015^*^ |
| **Left Lower Limb Muscle(kg)** | 8.7±2.1 | 9.0±1.8 | 8.7±2.1 | 8.1±2.4^a^ | 0.018^*^ |
| **ASMI (kg/m^2^)** | 7.6±1.4 | 7.9±1.2 | 7.7±1.3 | 7.0±1.8^ab^ | <0.001^*^ |
| **Sarcopenia (Yes)** | 69 (17.8%) | 9 (8.4%) | 41 (18.8%) | 19 (30.6%) | 0.001^*^ |

^a^ indicates that the difference was statistically significant compared to the low-risk group.

^b^ indicates that the difference is statistically significant compared to the medium-risk group.

**BMI:** body mass index; **ASMI:** appendicular skeletal muscle mass index.

^*^ P< 0.05.
