# Supplementary material for: Correlation analysis between body composition, serological indices and the risk of falls, and the receiver operating characteristic curve of different indexes for the risk of falls in older individuals
Source: Front Med (Lausanne). 2023 Jul 25;10:1228821. doi: 10.3389/fmed.2023.1228821 (PMC10409486; doi:10.3389/fmed.2023.1228821)
Supplement: Supplementary file 3 [file Table_3.DOCX]

Supplementary Material

Correlation Analysis Between Body Composition, Serological Indices and the Risk of Falls and **the receiver operating characteristic curve of different indexes for** the Risk of Falls in Older Individuals.

Kexin Zhang^1^, Yanmin Ju^1^, Di Yang^1^, Mengyu Cao^1^, Hong Liang^1^, Jiyan Leng^1*^

^1^Department of Cadre ward, The First Hospital of Jilin University, Changchun 130021, China

*** Correspondence:**Jiyan Leng
lengjy@jlu.edu.cn

# Supplementary Tables

**Table3** Comparison of serological indices among the low-risk, medium-risk, and high-risk groups

|  | **All** | **Low Risk Group** | **Medium Risk Group** | **High Risk Group** | **P** |
| --- | --- | --- | --- | --- | --- |
| **NE%** | 0.66±0.58 | 0.60±0.13 | 0.71±0.68 | 0.59±0.14 | 0.175 |
| **NE#(10^9/L)** | 3.87±1.84 | 3.83±1.76 | 3.87±1.86 | 3.96±1.91 | 0.895 |
| **HGB(g/L)** | 131.19±21.65 | 140.38±18.47 | 130.50±21.23^a^ | 117.74±20.91^ab^ | <0.001^*^ |
| **HCT(L/L)** | 0.40±0.05 | 0.41±0.05 | 0.41±0.05 | 0.38±0.05^ab^ | <0.001^*^ |
| **AST(U/L)** | 20.26±6.58 | 21.80±8.04 | 19.67±5.13^a^ | 19.68±7.90^a^ | 0.018^*^ |
| **ALT(U/L)** | 17.09±7.36 | 17.51±8.42 | 17.54±6.70 | 14.80±7.36^ab^ | 0.028 |
| **ALP(U/L)** | 131.16±17.4 | 63.00±15.46 | 184.46±18.4 | 63.44±14.82 | 0.315 |
| **ChE(U/L)** | 6921.1±2168.7 | 7254.1±2316.6 | 6801.8±2202.7 | 6765.7±1711.3 | 0.177 |
| **ALB(g/L)** | 36.79±5.10 | 37.91±3.94 | 37.41±4.02 | 32.69±7.73^ab^ | <0.001^*^ |
| **PA(g/L)** | 0.30±0.54 | 0.35±0.12 | 0.29±0.06 | 0.21±0.06 | 0.234 |
| **TC(mmol/L)** | 4.16±0.99 | 4.12±1.01 | 4.16±0.99 | 4.19±0.98 | 0.897 |
| **TG(mmol/L)** | 1.51±1.42 | 1.42±0.81 | 1.64±0.68 | 1.21±0.58^b^ | 0.084 |
| **HDL-C(mmol/L)** | 1.14±0.29 | 1.12±0.25 | 1.16±0.32 | 1.13±0.23 | 0.464 |
| **LDL-C(mmol/L)** | 2.38±0.76 | 2.34±0.72 | 2.40±0.79 | 2.33±0.71 | 0.696 |
| **FBG(mmol/L)** | 5.34±1.15 | 5.44±0.85 | 5.28±1.26 | 5.35±1.21 | 0.512 |
| **Anemia** |  |  |  |  | 0.007^*^ |
| **Normal** | 307（79.5%） | 95（88.8%） | 166（76.5%） | 46（74.2%） |  |
| **Mild** | 64（16.6%） | 12（11.2%） | 42（19.4%） | 10（16.1%） |  |
| **Moderate** | 15（3.9%） | 0（0.0%） | 9（4.1%） | 6（9.7%） |  |
| **Severe** | 0（0.0%） | 0（0.0%） | 0（0.0%） | 0（0.0%） |  |
| **Extremely Severe** | 0（0.0%） | 0（0.0%） | 0（0.0%） | 0（0.0%） |  |
| **Hypoproteinemia (Yes)** | 44（11..4%） | 10（9.3%） | 14（6.4%） | 20（32.3%） | <0.001^*^ |

^a^ indicates that the difference was statistically significant compared to the low-risk group.

^b^ indicates that the difference is statistically significant compared to the medium-risk group.

**NE%:** percentage of neutrophils; **NE#:** absolute neutrophil count; **HGB:** Hemoglobin, **HCT:** hematocrit; **AST:** aspartate aminotransferase; **ALT:** alanine aminotransferase; **ALP:** alkaline phosphatase; **ChE:** cholinesterase; **ALB:** Albumin; **PA:** prealbumin; **TC:** cholesterol; **TG:** triglyceride; **HDL-C:** high-density lipoprotein cholesterol; **LDL-C:** low-density lipoprotein cholesterol; **FBG:** fasting blood glucose.

^*^ P< 0.05.
