# Supplementary material for: Correlation analysis between body composition, serological indices and the risk of falls, and the receiver operating characteristic curve of different indexes for the risk of falls in older individuals
Source: Front Med (Lausanne). 2023 Jul 25;10:1228821. doi: 10.3389/fmed.2023.1228821 (PMC10409486; doi:10.3389/fmed.2023.1228821)
Supplement: Supplementary file 4 [file Table_4.DOCX]

Supplementary Material

Correlation Analysis Between Body Composition, Serological Indices and the Risk of Falls and **the receiver operating characteristic curve of different indexes for** the Risk of Falls in Older Individuals.

Kexin Zhang^1^, Yanmin Ju^1^, Di Yang^1^, Mengyu Cao^1^, Hong Liang^1^, Jiyan Leng^1*^

^1^Department of Cadre ward, The First Hospital of Jilin University, Changchun 130021, China

*** Correspondence:**Jiyan Leng
lengjy@jlu.edu.cn

# Supplementary Tables

**Table4** Ordinal logistic regression analysis of body composition and serological indices to predict the risk of falls among older adults in the low-, medium-, and high-risk groups

| **Indicators** | **B** | **SE** | **Wald** | **OR** | **95%CI** | ***P*** |
| --- | --- | --- | --- | --- | --- | --- |
| **Age(year)** | 1.089 | 0.295 | 13.637 | 2.971 | 1.667-5.296 | <0.001^*^ |
| **Education** | -0.643 | 0.316 | 4.155 | 0.526 | 0.283-0.975 | 0.042^*^ |
| **Cognitive Impairment（Yes）** | 1.317 | 0.487 | 7.318 | 3.732 | 1.438-9.689 | 0.007^*^ |
| **Malnutrition（Yes）** | 0.553 | 0.351 | 2.491 | 1.738 | 0.875-3.459 | 0.114 |
| **Increased Water Ratio(Yes)** | 0.529 | 0.415 | 1.626 | 1.697 | 0.753-3.831 | 0.202 |
| **Lower limb Edema (Yes)** | 1.336 | 0.425 | 9.900 | 3.804 | 1.655-8.741 | 0.002^*^ |
| **Decreased Skeletal Muscle Mass (Yes)** | 0.525 | 0.260 | 4.069 | 1.690 | 1.015-2.818 | 0.044^*^ |
| **Sarcopenia (Yes)** | 0.768 | 0.305 | 6.348 | 2.155 | 1.186-3.916 | 0.012^*^ |
| **Anemia** | 1.117 | 0.622 | 3.222 | 3.056 | 0.902-10.340 | 0.073 |
| **Hypoproteinemia (Yes)** | 0.545 | 0.371 | 2.155 | 1.725 | 0.833-3.572 | 0.142 |

**Dependent Variable:** the risk of falls.

**Independent Variable:** age, education, cognitive impairment, malnutrition, increased water ratio, lower limb edema, decreased skeletal muscle mass, anemia, and hypoproteinemia.

^*^ P< 0.05.
