# Supplementary material for: Correlation analysis between body composition, serological indices and the risk of falls, and the receiver operating characteristic curve of different indexes for the risk of falls in older individuals
Source: Front Med (Lausanne). 2023 Jul 25;10:1228821. doi: 10.3389/fmed.2023.1228821 (PMC10409486; doi:10.3389/fmed.2023.1228821)
Supplement: Supplementary file 5 [file Table_5.DOCX]

Supplementary Material

Correlation Analysis Between Body Composition, Serological Indices and the Risk of Falls and **the receiver operating characteristic curve of different indexes for** the Risk of Falls in Older Individuals.

Kexin Zhang^1^, Yanmin Ju^1^, Di Yang^1^, Mengyu Cao^1^, Hong Liang^1^, Jiyan Leng^1*^

^1^Department of Cadre ward, The First Hospital of Jilin University, Changchun 130021, China

*** Correspondence:**Jiyan Leng
lengjy@jlu.edu.cn

# Supplementary Tables

**Table 5** Correlation between different indexes and the occurrence of falls

|  | **AUC (95%CI)** | ***P*** | **Cutoff value** | **Sensitivity (%)** | **Specificity (%)** |
| --- | --- | --- | --- | --- | --- |
| **MMSE(score)** | 0.654(0.595-0.714) | <0.001^*^ | 19.33 | 62.65 | 56.68 |
| **Intracellular Water(L)** | 0.574(0.514-0.635) | 0.024^*^ | 10.16 | 58.87 | 51.29 |
| **Skeletal Muscle Mass(kg)** | 0.574(0.513-0.635) | 0.024^*^ | 7.67 | 57.91 | 49.76 |
| **Hemoglobin** | 0.663(0.602-0.723) | <0.001^*^ | 21.01 | 63.64 | 57.37 |

^*^ P< 0.05.
